# Disturbance and the Dynamics of Coral Cover on the Great Barrier Reef (1995–2009)

**DOI:** 10.1371/journal.pone.0017516

**Published:** 2011-03-10

**Authors:** Kate Osborne, Andrew M. Dolman, Scott C. Burgess, Kerryn A. Johns

**Affiliations:** Supporting Sustainable Use of Marine Biodiversity, Australian Institute of Marine Science, Townsville, Australia; Smithsonian's National Zoological Park, United States of America

## Abstract

Coral reef ecosystems worldwide are under pressure from chronic and acute stressors that threaten their continued existence. Most obvious among changes to reefs is loss of hard coral cover, but a precise multi-scale estimate of coral cover dynamics for the Great Barrier Reef (GBR) is currently lacking. Monitoring data collected annually from fixed sites at 47 reefs across 1300 km of the GBR indicate that overall regional coral cover was stable (averaging 29% and ranging from 23% to 33% cover across years) with no net decline between 1995 and 2009. Subregional trends (10–100 km) in hard coral were diverse with some being very dynamic and others changing little. Coral cover increased in six subregions and decreased in seven subregions. Persistent decline of corals occurred in one subregion for hard coral and Acroporidae and in four subregions in non-Acroporidae families. Change in Acroporidae accounted for 68% of change in hard coral. Crown-of-thorns starfish (*Acanthaster planci*) outbreaks and storm damage were responsible for more coral loss during this period than either bleaching or disease despite two mass bleaching events and an increase in the incidence of coral disease. While the limited data for the GBR prior to the 1980's suggests that coral cover was higher than in our survey, we found no evidence of consistent, system-wide decline in coral cover since 1995. Instead, fluctuations in coral cover at subregional scales (10–100 km), driven mostly by changes in fast-growing Acroporidae, occurred as a result of localized disturbance events and subsequent recovery.

## Introduction

There is widespread scientific agreement that coral reef ecosystems worldwide are being rapidly degraded [1], [2]. Substantial declines in coral abundance are thought to have occurred in most coral reef regions [3] and coral decline is frequently described as ongoing with the integrity and persistence of the reef system threatened by a number of different stressors [4]. Given the important ecosystem services of corals to reef ecosystems, including reef accretion and provision of habitat structure, accurately quantifying the dynamics of coral is important to both scientists and managers in understanding the causes and consequences of reef degradation.

Climate change is widely regarded as the single greatest threat to coral reef ecosystems. There are clear links between increases in ocean temperature and coral bleaching. Global patterns of coral loss [5] suggest that areas closest to urban centres are most degraded, implying that chronic stressors are compounding the effects of warming. Most studies on how climate change will impact coral reefs predict changes in the frequency or severity of existing stressors. There are, however, few studies that have directly compared the relative effects of different disturbances and data on the propensity, severity, or spatial patterns of impacts across large spatial scales. Severe storms can strip the entire substrate [6], [7], [8] and affect large reef tracts while less intense storms may affect only fragile growth forms [9] and promote fragmentation that leads to rapid regeneration [10]. *Acanthaster planci* predation, coral disease, and bleaching also have differential impacts depending on severity [11], [12], [13]. Understanding the patterns of change in coral cover in relation to different sources of disturbance should provide a broader understanding of risks to reef resilience.

Coral decline can result from disturbance that is too frequent or intense, or from depressed recovery due to stress or recruitment failure, as has been documented in the Caribbean [14]. Mass mortality of coral on the Great Barrier Reef (GBR) has been associated with *A. planci* [15], bleaching [16], disease [17] and storms [7], [18]. Recent research on disturbance and recovery of corals on the GBR has mostly examined responses to specific impacts such as bleaching [19], outbreaks of *A. planci* [20], [21], storms and cyclones [7], [22] or has documented changes in coral communities in relation to specific environmental gradients [23], [24], [25]. Globally, there are only 26 records for coral bleaching prior to 1982 and the scale and extent of bleaching on the GBR since 1998 is unprecedented [26]. Coral disease is an emerging stressor that was first recorded on the GBR in the early 1990s [17], [27]. Cyclone intensity is also predicted to increase in a warming climate and since 1995, three high intensity systems have crossed the GBR [28]. *A. planci* outbreaks peaked in 2003 but reefs on the southern GBR have been experiencing continuous *A. planci* predation since monitoring began [29]. Disturbances appear to be increasing in frequency and severity and it is not known whether coral growth will be able to keep pace with increased disturbance. Rapid recovery of disturbed reefs has been recorded with some reefs taking less than 10 years to recover their previous communities from low coral cover [18], [22]. However modelling studies based on current rates of disturbance and recovery predict long-term declines on both inshore and offshore reefs [30], [31].

Percent cover of live coral is by far the most widely used metric of coral reef condition and is universally used in studies that document coral reef decline and recovery across large spatial scales [3], [32], [33]. Determining regional trends in coral cover is difficult due to the large spatial and temporal scales involved. The variability and stochastic nature of disturbance events that shape reef communities mean that small scale studies can easily miss or over represent the impact of localised disturbance events. The data record for coral reefs prior to the 1980s is very limited. On the GBR, regional studies that include data prior to mid 1980's are all based on metadata and suggest reef condition has declined. Bellwood et al [4] show ongoing decline up to 2004, while Bruno et al [33] estimate there were substantial losses of coral just before the mid 80's. There are few reef regions where systematically collected large scale data are available. For the GBR, we are fortunate to have data from a dedicated large-scale monitoring program. Regional estimates of coral cover using the manta-tow method are available from the mid 80's and found average reef-wide coral cover across the GBR declined from 28.1% to 21.7% between 1986 and 2004 [34], a substantially lower figure than estimates made from metadata. Since 1992, the monitoring of permanent sites, designed specifically to assess change in coral cover, has avoided many of the short-comings associated with metadata and allows for a powerful analysis of coral cover change over time and multiple spatial scales.

The aims of this study were threefold. Firstly, to examine the patterns of change and estimate the extent of decline in total hard coral, Acroporidae, and non-Acroporidae coral on the GBR from 1995 to 2009 across multiple spatial scales. Secondly, to identify the agents of disturbance that have caused coral decline. Thirdly, assess the extent of recovery in coral cover after periods of decline. Our results indicate that, from 1995 to 2009, GBR-wide coral cover did not decline. Rather, there have been contrasting and uncorrelated temporal trends in coral cover at subregional scales (10–100 km), driven mostly by changes in fast-growing Acroporidae as a result of localized disturbance events, mainly *A. planci* predation and cyclones.

## Results

### Spatial and temporal trends in total hard coral cover, Acroporidae, and non-Acroporidae coral

**Figure 1:**
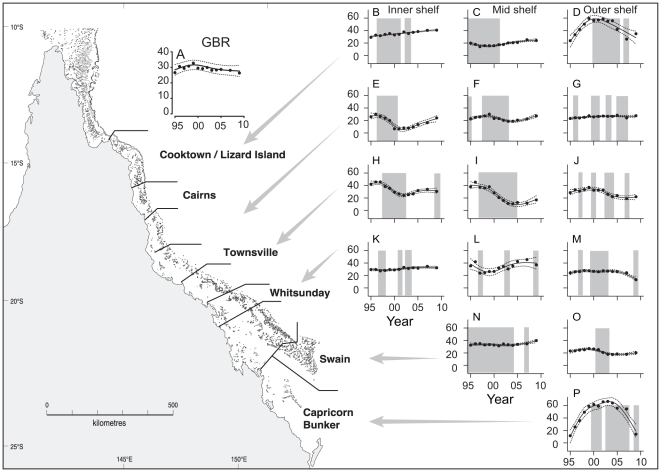
Temporal trends in percent cover of hard coral on the Great Barrier Reef (1995–2009). (A) Average coral cover for the whole GBR; (B–P) Average coral cover in each subregion. Dots and dashed lines show the average subregion temporal profile with 95% confidence intervals. For the whole GBR there was a non-significant linear trend of −0.27%, (−0.68, 0.14 95%CI) as a result of asynchronous increases and decreases in each subregion. The grey shaded area indicates periods of coral decline associated with disturbance.

**Figure 2:**
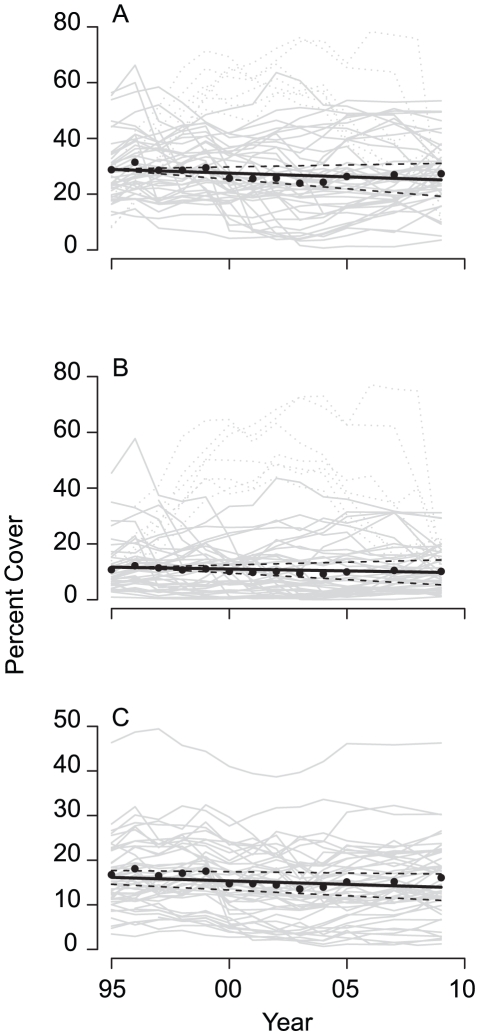
Linear trends in percent cover of hard coral, Acroporidae and non-Acroporidae families on the Great Barrier Reef (1995–2009). (A) Hard Coral, (B) Acroporidae coral, (C) Non-Acroporidae coral. Straight lines show the linear trend and confidence intervals (dashed lines). Individual reef profiles are in grey. Non-Acroporidae coral declined significantly (Table S1).

**Figure 3:**
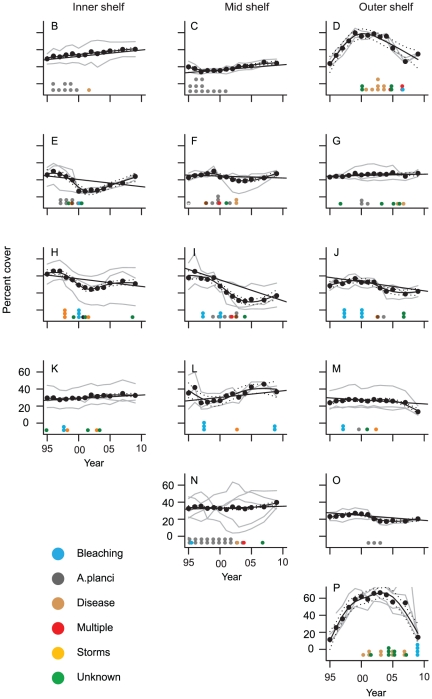
Temporal trends in percent cover of hard coral on the Great Barrier Reef (1995–2009). (B–P) Average annual coral cover in each subregion. Dashed lines show the subregion temporal profile and the straight lines show the average linear trend. Individual reef profiles are in grey. Disturbances associated with coral decline are represented by a dot for each reef where that type of disturbance occurred.

At the scale of the whole GBR, there was no net decline in hard coral cover between 1995 and 2009. Average cover was 30% over this period, peaking at 33% in 1999 and lowest at 27% in 1995 (Fig. 1A). However, this apparent overall stability was a product of variable and asynchronous increases and decreases in coral cover at the scale of subregions (Fig. 2A, Fig. 3, B–P). Coral cover varied dramatically in some subregions (e.g., Fig. 3D, P) while changing relatively little in others (e.g., Fig. 3C,G, K, N). The linear trend in total hard coral cover was positive for 6 subregions and negative for 7 (Table S1). For two subregions (Fig. 3D, P) the trend in total cover was clearly non-linear and linear trends were not assessed. Only one subregion (Fig. 3I) had a statistically significant linear trend, which was negative (Table S1).

Temporal trends in the cover of Acroporidae corals were, in most cases, similar to those for total coral cover (Fig. S1, Table S1). Overall, change in the cover of Acroporidae accounted for 68% of the change in total hard coral cover. The GBR wide trend in Acroporidae cover was also slightly negative (−0.13%, −0.47, 0.20 95%CI) per year but non-significant (Fig. 2B). At subregional scales, the cover of Acroporidae had qualitatively similar positive and negative trends to hard coral (Table S1). For both total hard coral and Acroporidae, reefs within subregions had similar temporal profiles in all but one subregion (Fig. 3N, Fig. S1N), where *A. planci* predation reduced coral cover on two surveyed reefs, while three reefs were unaffected.

At the scale of the whole GBR, the cover of non-Acroporidae declined −0.16% (−0.26, −0.06 95%CI) per year, which was statistically significant (Fig. 2C). At subregional scales, the cover of non-Acroporidae increased in 4 and decreased in 11 subregions (Fig. S1; Table S1). The cover of Acroporidae was also more variable over time than that of non-Acroporidae corals. The average coefficient of variation (CV) across all reefs was twice as large for Acroporidae (CV = 0.48) compared to non-Acroporidae (CV = 0.22).

### Impacts of different disturbance agents

**Figure 4:**
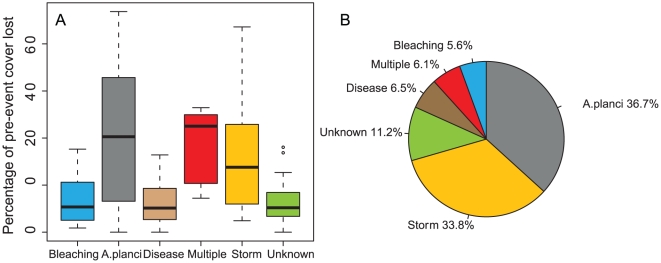
Prevalence and severity of disturbance events. *A.planci*, storms and multiple disturbances were associated with the greatest coral decline and the largest distribution of coral loss. Despite bleaching being widespread in 1998 and 2002, coral mortality was relatively low. (A) the loss in live coral cover as a percentage of the pre-event cover. (B) Relative magnitude of loss of pre-event cover summed for all reefs and years.

Storms and *A. planci* predation were the most important agents of decline, both in terms of their prevalence and severity (Table 1, Fig. 4). Storms and *A. planci* predation each accounted for approximately one third of coral loss and affected 24 and 23 individual reefs respectively (Table 1). *A. planci* outbreaks were somewhat more severe than storms, with reefs losing 42.2% of cover during *A. planci* outbreaks compared with 31.5% following storms (Table 1). Additionally, *A. planci* outbreaks typically affected reefs for 2–3 consecutive years or more, resulting in more reef-years of impact and a lower remaining absolute cover level than that following storms (Table 1).

**Table 1:** The effects of various agents of disturbance on live coral cover on the Great Barrier Reef from 1995 to 2009.

Disturbance type	Storms	*A.planci*	Disease	Multiple	Bleaching	Unknown
% of summed proportional loss	33.8	36.7	6.5	6.1	5.6	11.2
No. reefs affected	24	23	11	5	10	21
Reef-years of effect	32	71	22	6	12	29
Mean proportion of existing cover lost per event	−31.5	−42.2	−12.2	−36.6	−13.9	−12.9
Mean pre-event coral cover	38.4	32.2	53.0	43.2	36.9	39.8
Mean post-event coral cover	25.5	18.1	46.9	28.1	31.4	35.0

Bleaching and disease had far less impact than *A. planci* and storms in prevalence and severity. Eleven reefs were affected by disease, with a mean of 12.2% of pre-existing cover lost per outbreak, accounting for just 6.5% of total overall proportional losses (Table 1). Observed bleaching events were consistent with the overall pattern described for the GBR [35] with coral losses of 30–35% of the existing coral cover on the worst affected reef (Fig. 3H). However, while bleaching was widespread in 1998 and 2002 and locally severe on the southern GBR in 2006 [36] only ten of the sampled reefs had coral decline. Mean coral cover loss attributed to bleaching was 13.9%, just 5.6% of total losses across all reefs. Pre-event coral covers were similar for bleaching, *A. planci* and storm events (means of 37, 32, 38% respectively) but higher for disease, multiple and unknown agents of decline (means of 53, 43, 40% respectively) (Table 1).

Spatial patterns of disturbances shifted over the survey period from the inner and mid-shelf to the outer shelf. Most inshore and mid-shelf subregions had periods of continuous disturbance up to 2002 but have been in recovery phases since the mid 2000s (Fig. 1, Fig. 3). On outer shelf subregions disturbance has been more intermittent (Fig. 1). This was also the case in the Whitsunday sector across all shelf positions (Fig. 1K, L, M). Ten reefs had intense disturbance where coral cover declined to less than 10%, including two of the three reefs in the Townsville mid-shelf where there was a significant decline in coral (Fig. 3I). Coral cover less than 10% occurred on at least one reef in 7 subregions and included inner, mid and outer shelf reefs. Most reefs (8 of 10) had *A. planci* or *A. planci* plus other impacts. Storms were responsible for low coral cover on two outer shelf reefs.

On average there were 4 years between disturbances. Small disturbances (<5% annual change) to coral cover were common. Forty one reefs had between one and five small disturbances within the sampling period that were associated with all disturbance types. Moderate disturbances, defined as declines of between 5–10%, occurred on 27 reefs. Moderate declines were associated with all disturbance types. Large disturbances associated with declines of 10–17% occurred on 18 reefs and were associated with all disturbance types. Very large disturbances with coral declines of greater than 17% occurred on 13 reefs and were associated with *A. planci* predation, storms, and multiple disturbances. There was an average of six years between moderate disturbances and 11 years between large disturbances.

### Recovery from disturbance

**Figure 5:**
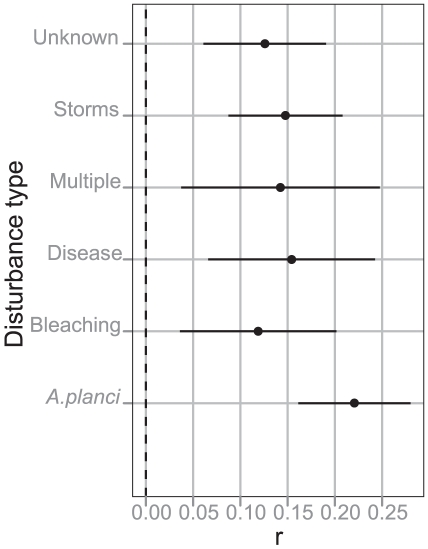
Intrinsic growth rates following different disturbance types. The intrinsic growth rate *r* estimated by fitting a logistic growth model to periods of coral recovery following disturbance events. Growth rates were similar following all disturbance types with the exception of *A. planci*. Carrying capacity *K* was estimated at 80.1%.

Disturbance type did not affect recovery rate with the exception of *A. planci*, after which estimated intrinsic growth rate was 0.22 (0.16, 0.28 95%CI) compared with an average of 0.14 (0.09, 0.19 95%CI) for all other disturbance types (Fig. 5). Average growth rate in disturbance free years was 3.9% (3.5,4.2 95%CI). The fastest recovery rates were observed on reefs dominated by tabulate *Acropora spp* (Fig. 3D, P). The seven reefs in these two subregions had low coral at the start of sampling in 1995, with all reefs increasing to 50–75% coral cover. The average growth rate during this period of increase, which lasted between 7 and 16 years, was between 5–11% per year with a maximum annual increase of 19% in one year. Growth periods at other reefs had mean values of 2–5%. The median length of recovery between disturbances was 5 years.

We identified 36 reefs where either coral cover did not decline or cycles of disturbance and recovery were completed. We found coral cover on 33% (12 reefs) of reefs did not decline, 57% (21 reefs) of reefs declined and recovered, and 8% (3 reefs) of reefs declined without recovery. Eleven reefs were excluded as having had recent disturbance or low starting values for coral cover and low recovery. Of the three reefs with declines, two reefs had additional disturbance before recovery from a prior disturbance was completed. One mid shelf reef (Reef 19131) in the Whitsundays lost coral due to a storm in 1997, and then coral bleaching in 2002 took coral cover to a new low before recovery had been completed. In 2009 coral cover had recovered to 72% of its maximum value of 60% coral cover. One outer shelf reef in the Townsville sector (Myrmidon) was recovering from the 2002 bleaching event when it was damaged by storms in 2007. In 2009 coral cover was 74% of its maximum of 39% coral cover. A number of reefs were still in recovery phases in 2009 and have not reached their pre-disturbance cover. The primary impact on the third reef, Havannah Island, was coral bleaching, which was followed by storms and sub-outbreak numbers of *A. planci*. A shift to macroalgae occurred and coral cover was still below 10% in 2010.

## Discussion

This study indicates that at the scale of the whole GBR there was no net decline in live hard coral cover between 1995 and 2009. Rather there have been contrasting and uncorrelated temporal trends in coral cover, driven mostly by Acroporidae corals, at subregional scales (10–100 km) resulting from localized disturbance events. Despite two category 5 cyclones and increased incidence of coral disease and bleaching since 1998, the level of hard coral cover throughout the system as a whole has changed little since 1995. We contend that 27–33% cover of hard coral represents a meaningful and accurate baseline range for average coral cover on the GBR since 1995. The patterns of temporal change within subregions were diverse but the overall pattern is of dynamic stability with the number of increases and decreases being similar both in number of subregions and numbers of reefs.

The limited data on coral cover prior to the mid-1980s suggest that coral cover on the GBR declined before our surveys began, and that we are reporting stability in a ‘shifted baseline’ [37], [38]. A shifted baseline is a likely outcome if disturbance is too frequent or intense, or if recovery is too slow [4], [33]. Data from elsewhere in the Pacific prior to the 1980's also suggest higher coral cover to what we report here [39]. Regional stasis has been reported for the Caribbean from 1982 to 2006 [40] whereas on the GBR there has been a small decline in coral cover since 1986 [34]. Estimates of coral cover from entire reef perimeters (using the manta-tow technique) found the GBR mean had declined from 28-22% between 1986 and 2004 [34]. The lower average from reef perimeter surveys as compared to our result is due to large areas of non-coral habitat (e.g., sandy back-reef lagoons) being included in estimates. The sites we sampled were located on the flank of the reef, an area of active reef accretion and good circulation. We would expect our results to be a ‘best case’ scenario for the GBR as a whole.

This study represents a robust baseline from which to assess future changes, but there is also an obvious need to know whether there is any evidence of changes in the frequency of disturbances. During the period 1995–2009 there were several cycles of the Southern Oscillation Index, which is an important driving factor of disturbance events on the GBR [28] but estimating changes in the periodicity of particular disturbance types is not possible with only 16 years of data. We have established a baseline for disturbance of every 4 years. The frequency of moderate (every 6 years) and large disturbances (>11 years) can be used for assessing relative disturbance pressure at a reef or for modelling changes in disturbance and response through time. The variability of coral decline associated with disturbances of each type indicates the complexity of factors influencing disturbance and response. These include disturbance history and level of coral cover, the type of coral community, as well as environmental factors such as exposure and circulation. A detailed understanding of the variability of these factors is needed to determine if current disturbance regimes are sustainable. The stability of the GBR average coral cover and overall balance of increases and decreases in coral cover at subregional scales suggests the disturbance intervals we have defined are meaningful in the current environment.

Since 1995, *A. planci* and storms impacted the largest number of reefs and caused the largest declines in coral cover on the GBR. Since regular monitoring of *A. planci* started in the mid-1980s the periodicity of *A. planci* outbreaks has been approximately 15 years on the central GBR [41], with outbreaks in the Townsville sector peaking in 1986 and 2003. Recovery of reef-wide coral cover between outbreaks was variable and model results suggest that the outbreak frequency is too high for adequate coral recovery between outbreaks [42], [43]. Since the most recent outbreaks, disturbance frequency was high with recovery most likely impaired by bleaching in 2002. This is especially likely on the Townsville mid-shelf where coral declined significantly (Fig. 3I).

Up to 2009, the majority of declines in coral cover were associated with particular disturbances. We attribute the relatively low contribution of coral disease and bleaching to coral declines to relatively low levels of stress (e.g., over-fishing and pollution) in the GBR system. Stressors may be more patchy and localised due to the low anthropogenic pressure relative to other reef regions and the dynamics of the reef matrix which promote water circulation [44]. The number of declines in coral cover associated with ‘unknown’ agents has increased since 2000, but even if these could be attributed to disease or bleaching, the magnitude of decline would still be low compared to *A. planci* and storms. Surveys in 1998 and 2002 identified large areas of reef where coral was bleached [35]. Our results indicate that mortality was not widespread or severe at depths of 6–9 meters. Since the last widespread bleaching event on the GBR in 2002, summer sea surface temperatures have been high in some locations. Both disease and bleaching have clear links with increased temperature and are likely to be important causes of chronic mortality on stressed reefs [45].

The long-term persistence of reefs requires coral communities to recover between episodic disturbance events. While our dataset is unique in its spatial and temporal coverage, 16 years is still a short period in which to document disturbance and recovery cycles, and the specific time frame influences our observations. For reefs where we were able to document cycles of disturbance and recovery, a high percentage (92%) either did not decline or declined and recovered. We found two reefs where additional disturbance interrupted recovery before the pre-disturbance coral cover was reached. Both reefs were back to 70% of their maximum in 2009 and increasing.

Recovery was similar across disturbance types except on reefs recovering from *A.planci* that had higher intrinsic growth rates. Similar results were found for other reef regions [46] and have been attributed to the maintenance of structural complexity following *A.planci*. While bleaching and disease also leave an intact skeleton, thermal stress associated with these disturbance types impairs reproductive success in *Acropora spp* [47], [48]. The fine-scale complexity of tabulate *Acropora* skeletons may provide protection from grazing in the early stages of growth when pressure can be intense (A. Thompson, pers comm.). Alternately the maintenance of fish diversity in habitats with higher structural complexity [9] may help create suitable substrate for coral recruitment.

Multiple disturbances were associated with declines on inner and mid-shelf reefs where coral cover dropped below 10%. The spatial and temporal aggregation of disturbances in the Cairns and Townsville sectors up to 2002 resulted in four inshore reefs and three mid-shelf reefs having low coral cover. Recovery was slow until 2007. On Cairns inshore reefs, shallower sites on the inshore reefs recovered to high coral cover sooner and had a higher proportion of *Acropora spp* driving coral cover change [29], [49]. Light limitation associated with turbidity is likely to be a factor inhibiting recovery on inshore reefs. Wet season rainfall has been high in recent years and has included flood events that have led to persistent turbidity in inshore environments [50]. At one reef (Green Island), recovery from *A. planci* outbreaks prior to 1995 was already poor on deeper sites and slow recovery may be due to recruitment failure [51]. On Havannah Island, a Townsville inshore reef, persistent macroalgae dominance, indicative of a ‘phase shift’, is possibly due to low diversity and abundance of herbivorous fishes (e.g., Acanthurids and Siganids) that can prevent the establishment of macroalgae [52]. A better understanding of disturbance risk and recovery potential is required to better manage reefs at local scales. There is substantial knowledge of individual risks for bleaching, cyclones, disease and *A. planci*. Areas least at risk of multiple or intense events should be identified, especially on the mid- and outer shelf where anthropogenic effects not related to climate change should be minimal.

It is not known to what extent low coral cover compromises ecosystem functioning, but there are indications that the amount of coral cover influences other species either directly or indirectly. While several studies have found declines in fish diversity when coral declines [53], [54], fish counts from AIMS survey reefs indicate that there was no long term loss of fish diversity or evenness, though fish abundance declined following loss of coral cover [55]. The shift from coral to macroalgae at Havannah Island has not resulted in fish species loss, but low starting values for species diversity and abundance may have been a factor in the long-term persistence of macroalgae [52]. Storms destroy the physical substrate, which has more severe consequences for fish abundance and diversity [56]. *A. planci*, bleaching and disease have less effect on fish populations, presumably due to the maintenance of topographic complexity [22], but see [21]. The effects of Acroporidae loss are well documented for specialist coral feeders [57] but the wider consequences to reef communities are not well known. With the exception of Havannah Island, all reefs that had low coral cover were in recovery phases in 2009, suggesting critical ‘tipping points’ have not been exceeded [58].

Climate change is expected to cause changes in relative abundance of species due to differential mortality and recovery rates [2]. The susceptibility of Acroporidae (and especially *Acropora* spp) to *A. planci* [20], bleaching [59], and disease [17] is well documented. Only one subregion had a significant decline in Acroporidae, suggesting that on most reefs, recruitment, growth and mortality are keeping up with the recent disturbance regime. For non-Acroporidae families, 11 of the 15 subregions had declines, three of which were statistically significant. All but one inshore subregion and all outer shelf subregions had negative trends for non-Acroporidae. Persistent declines are a ‘red flag’ for managers and researchers to elucidate where functional failures are occurring. The pressures on inshore reefs of the GBR have been well documented [60], [30] with existing research suggesting coral bleaching and elevated nutrients are adding unsustainable pressure to inshore reefs [61], [62]. Our results suggest that outer shelf reefs are also at risk due to intense disturbance pressure in recent years and large disturbance size, both spatially and in intensity of storms. For slower growing coral, evidence suggests that the current disturbance regime is unsustainable. Ecological health indices calculated from species richness estimates on GBR reefs found that species loss was a feature of depauperate coral communities, rather than a shift in community composition [23]. Loss or decline of less tolerant species would be consistent with our results and needs further investigation.

In conclusion, precise estimates of coral cover from a dedicated monitoring program revealed that system-wide coral cover changed very little on the GBR between 1995 and 2009. Although coral cover averaged 29% across the whole GBR, previous studies indicate that coral cover was higher prior to when our surveys began. Nonetheless, there appears to be no evidence of continued system-wide decline since 1995. During this 16 year period, storms and *A. planci* predation had the largest impact on coral cover, especially at subregional scales (10–100 km), in terms of reefs affected, summed coral lost at all reefs, and amount of decline at individual reefs. The impact of bleaching and coral disease, to date, was not severe on our sites. There are a number of factors however, that suggest that the current disturbance regime may not be sustainable. One inshore reef, for example, had a phase shift from hard coral to macroalgae, similar to that which has occurred at much larger scales in the Carribbean [63]. Corals with less capacity for growth and recruitment than Acroporidae had widespread negative trends. However, the abundance of Acroporidae species and relatively low anthropogenic agents of disturbance appears to place the GBR in a healthier state than the global average.

## Methods

This study was approved as part of ongoing research of the Australian Institute of Marine Science. Data were collected under Great Barrier Reef Marine Park Authority Permit G06/19994.1

### Sampling

Coral communities were surveyed annually between 1995 and 2009, on 47 reefs in six latitudinal sectors (Cooktown-Lizard Is, Cairns, Townsville, Whitsunday, Swain and Capricorn-Bunker) and in three positions on the continental shelf (inshore, mid-shelf and outer shelf) of the GBR. Each combination of latitudinal sector and continental shelf position is referred to as a ‘subregion’. There are no substantial reefs inshore in the Swains sector or in inshore and mid-shelf positions in the Capricorn-Bunker sector. Between two and five (usually three) reefs were surveyed in each subregion. Three sites on the north-east flank of each reef were surveyed. Each site consisted of five 50 m transects at depths between 6 m and 9 m that were marked with steel rods. Percent cover of live hard coral was estimated from a randomly selected sequence of images taken along the transects using a point-sampling technique in a quincunx pattern [64]. Corals were identified to a minimum taxonomic resolution of family and to genus where possible. Only 29 reefs were surveyed in the first two years and only 10 were surveyed in the 14^th^ and 16^th^ years due to changes in the scope of the monitoring program.

### Analyses

#### Spatial and temporal trends in total hard coral cover, Acroporidae, and non-Acroporidae

Where appropriate, linear trends in percent live hard coral cover were estimated using mixed-effects linear models with random effects to account for the repeated observations on reefs. Models were built in R (R Development Core Team 2008) with the NLME package [65], [66]. The GBR-wide trend in coral cover was estimated using a repeated-measures design with a fixed linear time parameter and random time and intercept effects for reefs. Trends for individual subregions were estimated using a model with a fixed linear time parameter for each subregion and random time and intercept effects for reefs. The addition of further random effects to model the spatial arrangement of reefs was explored but did not substantially influence the fitted parameters or the overall goodness of fit.

#### Identifying agents of disturbance

Declines in coral cover were attributed to *A. planci* predation, storm and cyclone damage, bleaching, and disease. The attribution of coral decline to a particular agent was based on evidence gathered by trained and experienced personnel during manta tow and SCUBA surveys [67]. Each of the four disturbance agents have distinctive and identifiable effects on corals. For example, the presence of *A. planci* and feeding scars indicated *A. planci* predation on live coral, while dislodged and broken coral indicated storm damage[7]. Coral bleaching also has distinctive effects on live coral compared to disease [67]. If no evidence was found, the agent of decline was recorded as unknown. If evidence of more than one agent was present, the cause was recorded as multiple. Although correlative, this approach represents our best estimate of the proximate causes of coral decline and is consistent with approaches used in other studies [7] [20]. It is of course possible that other chronic and sublethal disturbances occurred that are not reflected in total coral cover.

#### Assessing the impacts of different disturbance agents

The relative importance of each disturbance agent was assessed by comparing their prevalence and severity. Prevalence was assessed by counting the number of reefs affected and the number of reef-years of impact for each agent. For example, if a reef had bleaching for two years, then reef-years is recorded as two for that reef. Severity was estimated as the decline in the percent cover of live coral from pre-event cover. Since absolute loss in coral cover is positively correlated with the initial coral cover, coral losses were expressed as the proportion of pre-existing cover. Individual storms and mass bleaching events last from a few hours to a few weeks. Outbreaks of *A. planci* or diseases can cause coral mortality at a reef over several years. Where a disturbance lasted multiple years, cover loss was the total change in cover during that period as a proportion of the initial cover. The overall importance of each agent of decline was determined by summing the proportional losses across reefs and years. This sum of proportional losses from individual events gives a good representation of the relative importance of the agents and should not be biased by the sequence in which impacts occur. However, proportional coral loss may still be related to pre-event cover, as the highest cover values may be associated with more developed coral structures that, depending on the growth form of the corals, can be more susceptible to wave damage [68]. Accordingly, pre-event levels of coral cover were also examined to see whether they differed across disturbance types. Mean rates of change for hard coral were estimated from annual rates of change from each reef and year. Disturbance frequency was estimated from single year impacts. Small, moderate and large disturbances were defined by percentiles of the mean rate of annual coral decline (50^th^  = 5%, 75^th^ = 10%, 90^th^ = 17%). Change under 2% was excluded because this is within the range of sampling error [69].

#### Recovery from disturbance

We used the same criteria as Connell [56] to look at how many reefs have declined or recovered. Regression trees [70] were used to identify periods of consistent increase or decrease in hard coral cover, constrained in a way so that years in each grouping must be a sequence (e.g., years 2, 3 and 4 can be grouped but 2, 3 and 7 cannot). Tree sizes (number of periods of consistent change identified) were determined by cross-validation using data from the three sites at each survey reef.

If coral cover at a reef decreased proportionally by 33% from its pre-disturbance cover, a decline was recorded. If coral cover recovered to at least 50% of its pre-disturbance level it was recorded as recovered. Reefs that had recent disturbances, or had low starting values and had not recovered to the GBR average of 29% were excluded. To determine if disturbance type influenced subsequent recovery a logistic growth model was fitted to individual annual changes in total coral cover for 5 years post disturbance, allowing the intrinsic growth rate to vary according to the previous disturbance type. The logistic model was fit as a non-linear mixed effects model using the NLME package for R, with fixed effects on the intrinsic growth rate, *r,* according to previous disturbance type and a random effect on growth rate at the level of individual reefs.

## Supporting Information

Figure S1 **Temporal trends in percent cover of Acroporidae and non-Acroporidae on the Great Barrier Reef (1995–2009).** Average annual cover of Acroporidae and non-Acroporidae coral in each subregion (B–D). Straight lines show the average linear trend. Disturbances associated with coral decline are represented by a dot for each reef where that type of disturbance occurred, as in Figure 3.
(EPS)

Table S1 Linear trends in coral cover (1995–2009).
(DOC)
