# Correction: Disturbance and the Dynamics of Coral Cover on the Great Barrier Reef (1995–2009)

**DOI:** 10.1371/journal.pone.0099742

**Published:** 2014-06-02

**Authors:** The PLOS ONE Staff

The color coding of the categories in Figure 3 is incorrect. The authors have supplied a corrected version of Figure 3 below.

**Figure 3:**
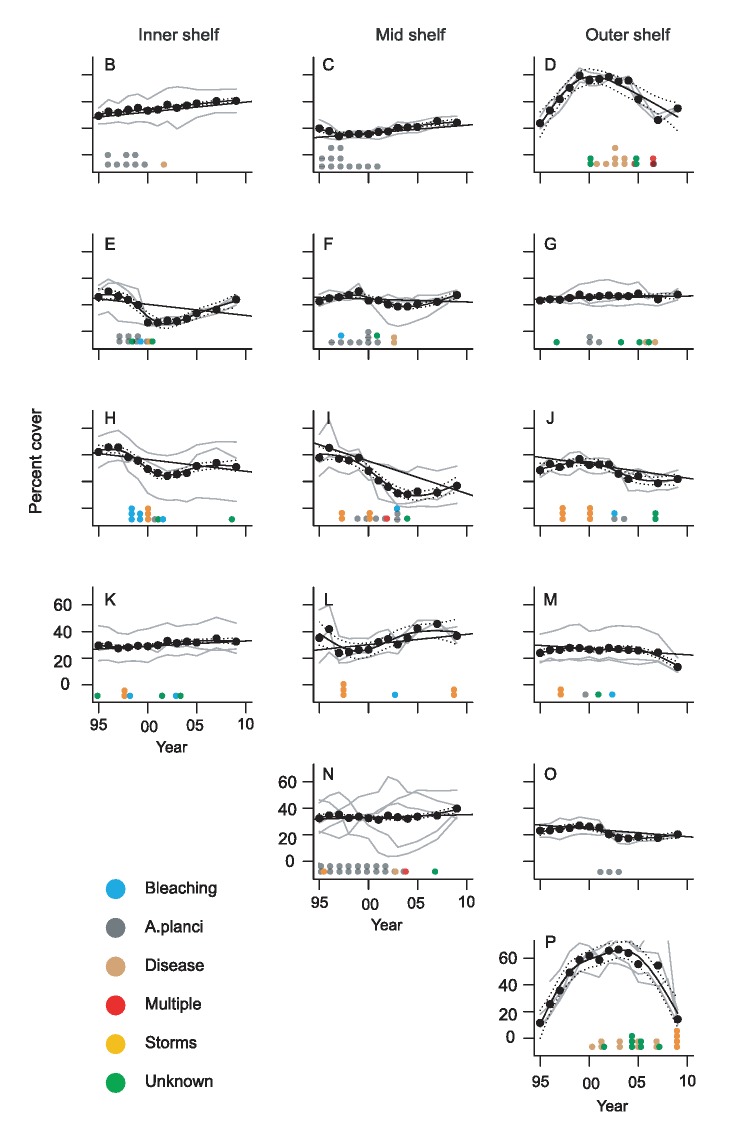
Temporal trends in percent cover of hard coral on the Great Barrier Reef (1995–2009). (B–P) Average annual coral cover in each subregion. Dashed lines show the subregion temporal profile and the straight lines show the average linear trend. Individual reef profiles are in grey. Disturbances associated with coral decline are represented by a dot for each reef where that type of disturbance occurred.
