# Supplementary material for: Disturbance and the Dynamics of Coral Cover on the Great Barrier Reef (1995–2009)
Source: PLoS One. 2011 Mar 10;6(3):e17516. doi: 10.1371/journal.pone.0017516 (PMC3053361; doi:10.1371/journal.pone.0017516)
Supplement: Table S1 — Linear trends in coral cover (1995–2009). (DOC) [file pone.0017516.s002.doc]

Table S1. Linear trends in coral cover (1995-2009)

A.

|  | **Average cover** | **GBR-wide trend** | **(95% CI)** | **t** | **p** |
| --- | --- | --- | --- | --- | --- |
| All hard coral | 27.01 | -0.27 | (-0.68, 0.14) | -1.28 | 0.199 |
| Acroporidae | 10.72 | -0.13 | (-0.47, 0.20) | -0.79 | 0.429 |
| Non-Acroporidae | 15.04 | -0.16 | (-0.26, -0.06) | -3.05 | 0.002 |
|  |  |  |  |  |  |
| B. |  |  |  |  |  |
| **Subregion** | **Average**  **cover** | **Trend**  **Hard Coral** | **Trend**  **Acroporidae** | **Trend  Non -Acroporidae** | |
| B | 35.85 | 0.75 | 0.47 | 0.28 | |
| C | 19.23 | 0.61 | 0.21 | *0.39 | |
| D | NA | NA | NA | -0.29 | |
| E | 16.19 | -0.83 | -0.53 | -0.30 | |
| F | 23.28 | -0.22 | -0.06 | -0.17 | |
| G | 25.83 | 0.15 | 0.26 | -0.11 | |
| H | 32.11 | -0.89 | -0.75 | -0.10 | |
| I | 23.15 | *-2.44 | *-1.88 | *-0.56 | |
| J | 27.75 | -1.05 | -0.48 | *-0.57 | |
| K | 30.91 | 0.40 | 0.43 | -0.03 | |
| L | 34.05 | 0.77 | 0.77 | 0.01 | |
| M | 24.91 | -0.47 | -0.17 | -0.30 | |
| N | 34.07 | 0.22 | 0.16 | 0.06 | |
| O | 21.54 | -0.58 | -0.23 | -0.35 | |
| P | NA | NA | NA | *-0.29 | |

Trends refer to rates of percent cover per year. A. Average cover and trends for whole GBR B. Average cover and trends in each subregion. Codes for subregions are referenced in Figure 1. * = significant trend with p< 0.05, NA=not tested due to non-linear temporal profile)
